# Significance of the NOR1-FOXA1/HDAC2-Slug regulatory network in epithelial-mesenchymal transition of tumor cells

**DOI:** 10.18632/oncotarget.7778

**Published:** 2016-02-27

**Authors:** Wei Wang, Mei Yi, Shengnan Chen, Junjun Li, Guo Li, Jianbo Yang, Pan Zheng, Haijing Zhang, Wei Xiong, James B. McCarthy, Guiyuan Li, Xiaoling Li, Bo Xiang

**Affiliations:** ^1^ Hunan Provincial Cancer Hospital and Cancer Hospital Affiliated to Xiangya Medical School, The Central South University, Changsha, Hunan 410013, China; ^2^ Cancer Research Institute, Xiangya School of Medicine, The Central South University, Changsha 410078, China; ^3^ Department of Dermatology, Xiangya Hospital, The Central South University, Changsha, 410008, Hunan, China; ^4^ Department of Otolaryngology Head and Neck Surgery, Xiangya Hospital, The Central South University, Changsha, 410008, Hunan, China; ^5^ Department of Pathology, Affiliated Hospital of Jining Medical University, Jining, 272029, Shandong, China; ^6^ Department of Laboratory Medicine and Pathology, Masonic Cancer Center, University of Minnesota, Minneapolis, Minnesota 55455, USA

**Keywords:** epithelial-mesenchymal transition, nasopharyngeal carcinoma, FOXA1, NOR1

## Abstract

The epithelial-mesenchymal transition (EMT) process is believed to play a crucial role in nasopharyngeal carcinoma (NPC) progression, a squamous cell carcinoma of the head and neck with the tendency to metastasize early. At present, much attention has been given to the inducer of EMT involved in NPC progression, while antagonists have been less intensively characterized. In this study, unbiased analysis of EMT-associated gene expression patterns was performed using data mining of global gene expression profiles derived from NPC samples, leading to the successful identification of NOR1, FOXA1, and Slug, all of which showed aberrant expression during NPC progression. The effect of tumor suppressor NOR1 on Slug-induced NPC cells during the EMT process was investigated by use of ectopic expression and RNA interference methods. The molecular mechanisms underlying the tumor-suppressing effect of NOR1 on Slug-induced EMT were thought to be dependent on the cooperation of NOR1 with the FOXA1-HDAC2 complex. We also showed that FOXA1 and HDAC2 bind the *slug* promoter and directly repress its transcription. Our data revealed a previously unrecognized role of the NOR1-FOXA1/HDAC2-Slug network in the regulation of the EMT process and aggressiveness of NPC.

## INTRODUCTION

Nasopharyngeal carcinoma (NPC) is a non-lymphomatous, squamous-cell carcinoma derived from malignant transformation of mucosal epithelial cells in the nasopharyngeal cavities [1]. NPC is rare in most parts of the world but has especially high incidence in Southern China and Southeast Asia [2]. NPC characteristically exhibits an early tendency to locally spread to the parapharyngeal space at an early stage [3]. According to one prospective study, cervical lymphadenopathy occurred in 204 of 271 (75.3%) consecutive patients with newly diagnosed NPC [4]. About 18% of non-metastatic NPC patients eventually develop isolated distant metastasis irrespective of a successful locoregional treatment [5]. Consequently, distant metastasis continues to be considered a major cause of treatment failure and death from NPC. At present, control of regional spread and distant metastasis of NPC remains a difficult and challenging problem.

The epithelial to mesenchymal transition (EMT) refers to loss of epithelial features and a shift towards a mesenchymal phenotype. Epithelial-derived carcinoma undergoes EMT which is often accompanied by a loss of contact inhibition and increased cell motility [6, 7]. The EMT process is thought to be implicated in tumor invasion and metastasis [8]. Diverse signal transduction cascades contribute to the process of EMT. TGF-β signaling has been shown to play an important role in EMT [9]. However, only a few murine cell lines and mouse models have been found to undergo TGF-β1-mediated complete EMT, although many cancer cell lines have shown response to TGF-β1, as evidenced by Smad2 phosphorylation induced by TGF-β1 [10]. NPC cell lines have also lost sensitivity to the growth-inhibitory effects of TGF-β1, although they showed evidence of functional TGF-β1/Smad signal transduction [11]. Thus, there may be alternative mechanisms for the EMT process in NPC progression.

Several transcription factors, including Snail1, Slug and Twist1, were initially identified as dominant inducers in the EMT process [12]. These EMT inducers are thought to function in a redundant manner; however, several recent studies suggest unique functions for Slug [13]. Aberrant overexpression of *snail* and the Twist protein has been reported to occur in the late stages of NPC, and has been associated with tumor aggressiveness [14, 15]. Whether Slug contributes to NPC progression remains to be elucidated. On the other hand, except for the initially identified EMT inducers mentioned above, other unknown transcription factors could also be involved [16]. The forkhead transcription factor FOXA1 is thought to be critical for both early embryonic development and late or end stage epithelial differentiation [17, 18]. Several pilot studies suggested that FOXA1 is intensively involved in the EMT process in pancreatic and lung cancers [18, 19]. However, the precise role of FOXA1 in cancer development is controversial [20]. Whether FOXA1 is involved in the EMT process and aggressiveness of NPC remains unknown.

The oxidored-nitro domain containing protein 1 gene (NOR1; also called organic solute carrier partner 1, or OSCP1) is a tumor suppressor gene (TSG) often silenced by DNA hypermethylation in NPC tissues and hematological malignancies [13, 21–25]. Another previous study showed that exogenously expressed NOR1 protein at a physiological level in NPC cells suppressed the EMT process as evidenced by induction of epithelial cytokeratin but downregulation of mesenchymal vimentin [26]. NOR1 mediation of the mesenchymal to epithelial transition (MET) process is associated with decrease of Slug but not Snail1. Despite these findings, little is known regarding the mechanisms underlying the influence of NOR1 on the MET process and NPC aggressiveness.

Microarray-based gene expression profiling enabled us to identify the key players modulating the EMT process during NPC progression in an unbiased fashion. In this study, we firstly analyzed the mRNA levels of EMT-associated genes by data mining a public NPC GEO data set, GSE12452, which contains 31 NPC and 10 normal nasopharyngeal tissue samples [27]. This unbiased analysis revealed that aberrantly high expression of Slug and low expression of NOR1 and FOXA1 occurs during NPC progression. Interestingly, NOR1 mRNA levels showed inverse correlation with those of Slug. Subsequent immunohistochemical staining further confirmed the alteration of these three proteins during NPC progression. We show next that NOR1 suppressed Slug-induced EMT and NPC aggressiveness. NOR1-mediated Slug inhibition in NPC cells is accompanied by the disturbance of Slug-associated histone-3-lysine-9 (H3K9) acetylation and tri-methylation, which is dependent on FOXA1 and histone acetyltransferase (HDAC)2. We further showed that FOXA1 binds to the *slug* promoter and represses its transcription. HDAC2 is responsible for de-acetylation of Slug-associated H3K9 and repression of *slug* transcription. Our data revealed a novel, unrecognized role of the NOR1-FOXA1/HDAC2-Slug network in regulating the EMT process and NPC aggressiveness.

## RESULTS

### Unbiased analysis of differential expressed EMT associated genes in NPC tissues

**Figure 1:**
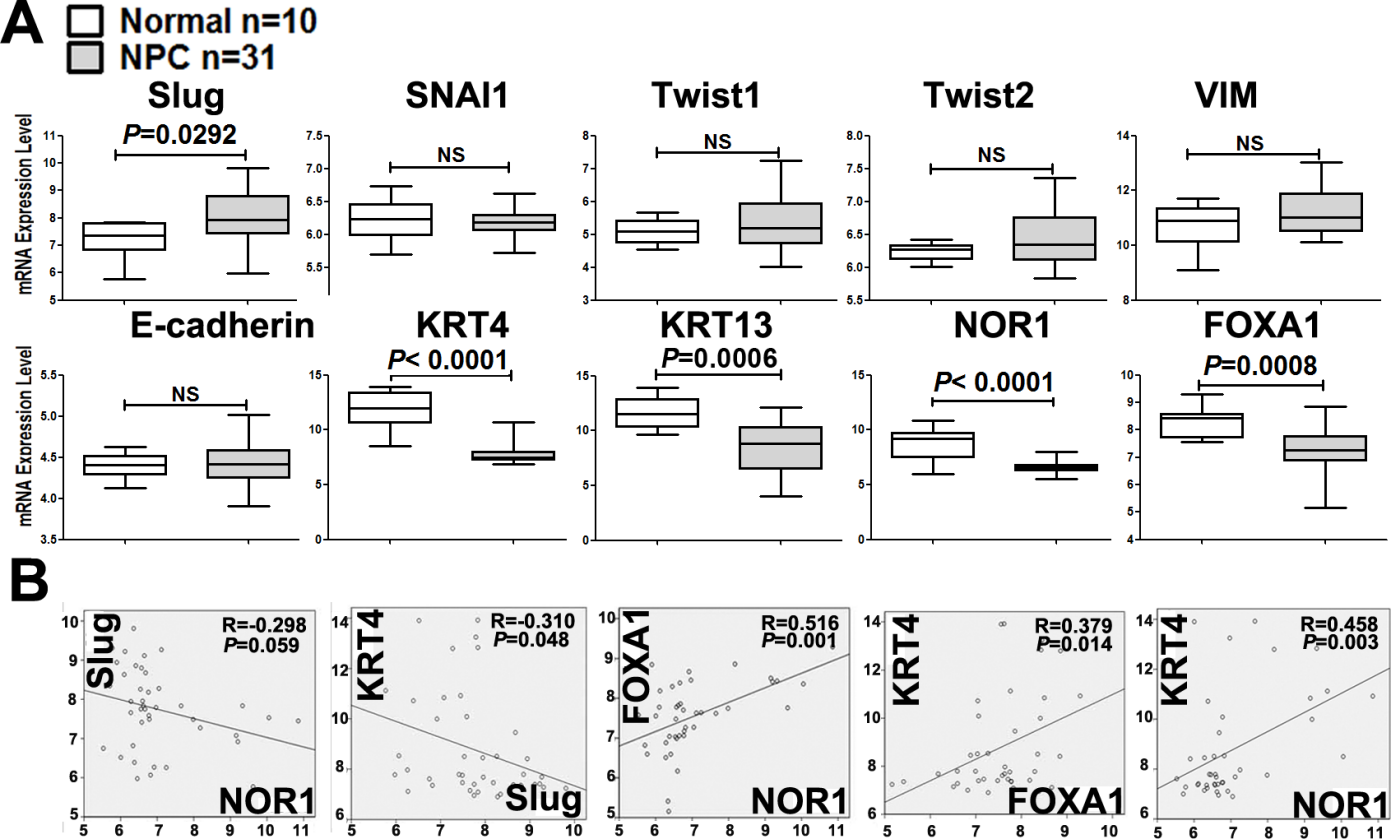
Unbiased analysis of EMT-associated gene mRNA levels by data mining of the NPC GEO dataset (**A**) Box plot showing the mRNA levels of EMT-associated molecules in NPC tissues. These data were collected from the global gene expression profile data set GSE12452, which contains 31 NPC and 10 normal nasopharyngeal tissue samples examined with a Human Genome U133 Plus 2.0 Array (HG-U133 Plus 2) from Affymetrix. (**B**) Correlations between differentially expressed EMT-associated genes in NPC. The levels of Slug mRNA were shown to be inversely correlated with keratin 4 and NOR1 mRNA levels. NOR1 mRNA levels were shown to be positively correlated with keratin 4 and FOXA1.

Firstly, we analyzed EMT-associated gene expression levels using microarray data collected from global gene profiling (GEO) dataset GSE12452, which contains 31 NPC and 10 normal nasopharyngeal tissue samples. The mRNA levels of NOR1, FOXA1, Slug, keratin 4 and keratin 13 were collected from GEO dataset GSE12452. Slug mRNA levels sharply increased in NPC samples, as compared to the levels in their healthy counterparts. However, the mRNA levels of three other EMT inducers, including Snail1, Twist1, and Twist2, in addition to those of E-cadherin and vimentin, remained unchanged between NPC samples and healthy nasopharyngeal tissue samples (Figure 1A). This implies that Slug might play a pivotal role in driving the EMT process during NPC tumorigenesis.

We also analyzed the mRNA levels of other EMT-regulating candidates during NPC progression and found that mRNA levels of NOR1, FOXA1, keratin 4, and keratin 13 were significantly lower in NPC tissue samples than in their normal counterparts (Figure 1A). Interestingly, there was an inverse correlation between Slug and keratin 4 mRNA levels (*R* = −0.310, *P* < 0.05) (Figure 1B). The level of Slug mRNA was found to be inversely correlated with NOR1 mRNA levels, although this difference was marginally significant (*R* = −0.298, *P* = 0.059) (Figure 1B). We also found the levels of NOR1 mRNA to be positively and readily correlated with FOXA1 (*R* = 0.516, *P* < 0.001) and keratin 4 (*R* = 0.458, *P* < 0.05) (Figure 1B). These data suggest that NOR1, FOXA1 and keratin 4 might be involved in Slug-induced EMT.

**Figure 2:**
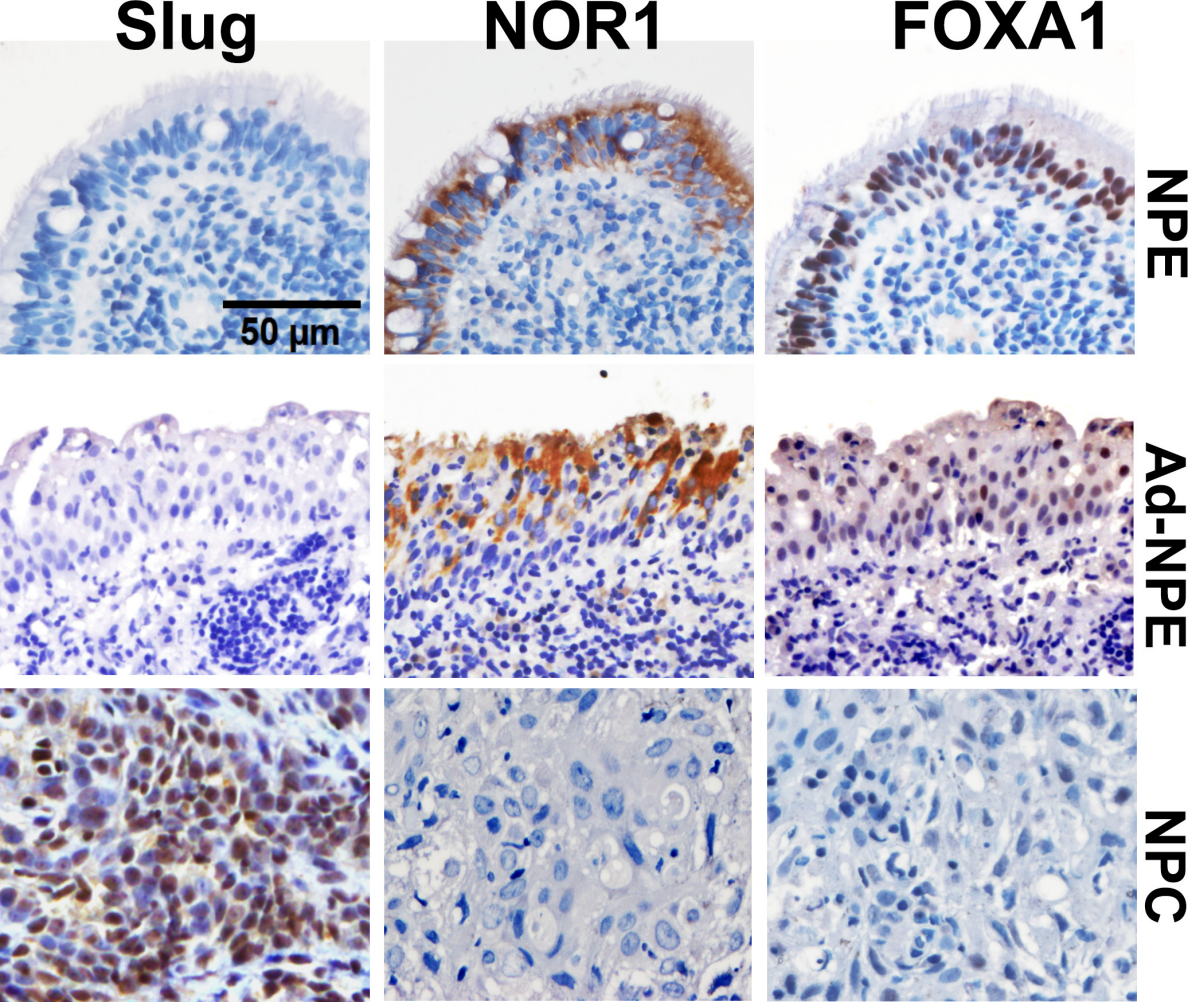
Lost expression of NOR1 and FOXA1 protein and increased immunoreactivity of slug protein in NPC cells Intensive nuclear immunohistochemical staining for Slug protein was found in NPC samples, while only weak staining for Slug protein was found in NPE or Ad-NPE cells. In contrast, intensive cytoplasmic immunohistochemical staining for NOR1 and intensive nuclear immunohistochemical staining for FOXA1 were found in NPE or Ad-NPE cells, but expression of NOR1 and FOXA1 proteins was lost in NPC samples.

We also quantified NOR1, FOXA1 and Slug protein levels in five inflammatory nasopharyngeal epithelium (NPE) samples, 26 non-cancerous nasopharyngeal epithelium adjacent to NPC (Ad-NPE) and 33 NPC tissue samples by immunohistochemical staining. Intensive nuclear immunohistochemical staining for Slug protein was observed in NPC samples, but negative or very weak in inflammatory NPE and Ad-NPE samples (Figure 2). In contrast, intensive cytoplasmic immunohistochemical staining of NOR1 and intensive nuclear immunohistochemical staining of FOXA1 protein were observed in NPE and Ad-NPE, whereas decreased immunoreactivity for both NOR1 and FOXA1 was observed in NPC cells (Figure 2). The immunohistochemical staining results are summarized in Table 1. Surprisingly, neither Slug nor NOR1 or FOXA1 protein levels showed any association with the status of lymph nodes metastasis. However, the expression of Slug protein was found to be associated with the clinical stage of NPC patients (*P* = 0.044, Table 1), while FOXA1 protein levels were only marginally associated with the lymph node metastasis of NPC patients (*P* = 0.065, Table 1).

**Table 1:** Expression of Slug, NOR, and FOXA1 proteins in NPC samples and their association with clinicopathological data of NPC patients

	Slug(+/−)	NOR1(+/−)	FOXA1 (+/−)
**Histological types**			
NPE (*n* = 5)^a^	0/5	5/0	5/0
Ad-NPE (*n* = 26)^b^	7/19	25/1	11/15
NPC (*n* = 33)^c^	25/8	2/31	5/28
*P*^ac^	0.001*	0.000*	0.000*
*P*^bc^	0.000*	0.000*	0.020*
**Clinical Stage**			
I - II (*n* = 11)	6/5	0/11	3/8
III-IV (*n* = 22)	19/3	2/20	2/20
*P*	0.044*	0.302	0.170
**lymph node metastasis**			
YES (*n* = 19)	15/4	2/17	1/18
NO (*n* = 14)	10/4	0/14	4/10
*P*	0.618	0.210	0.065
**Age**			
< 50 (*n* = 20)	17/3	2/18	2/18
≥ 50 (*n* = 13)	8/5	0/13	3/10
P	0.124	0.239	0.306
**Gender**			
Female (*n* = 6)	6/0	0/6	2/4
Male (*n* = 27)	19/8	2/25	3/24
*P*	0.126	0.492	0.170

NPE: Inflammatory nasopharyngeal epithelium

Ad-NPE: non-cancerous nasopharyngeal epithelium adjacent to NPC

NPC: nasopharyngeal carcinoma

(+), immunostaining positive. (−), immunostaining negative

### NOR1 suppresses the slug-driven EMT process in NPC cells

**Figure 3:**
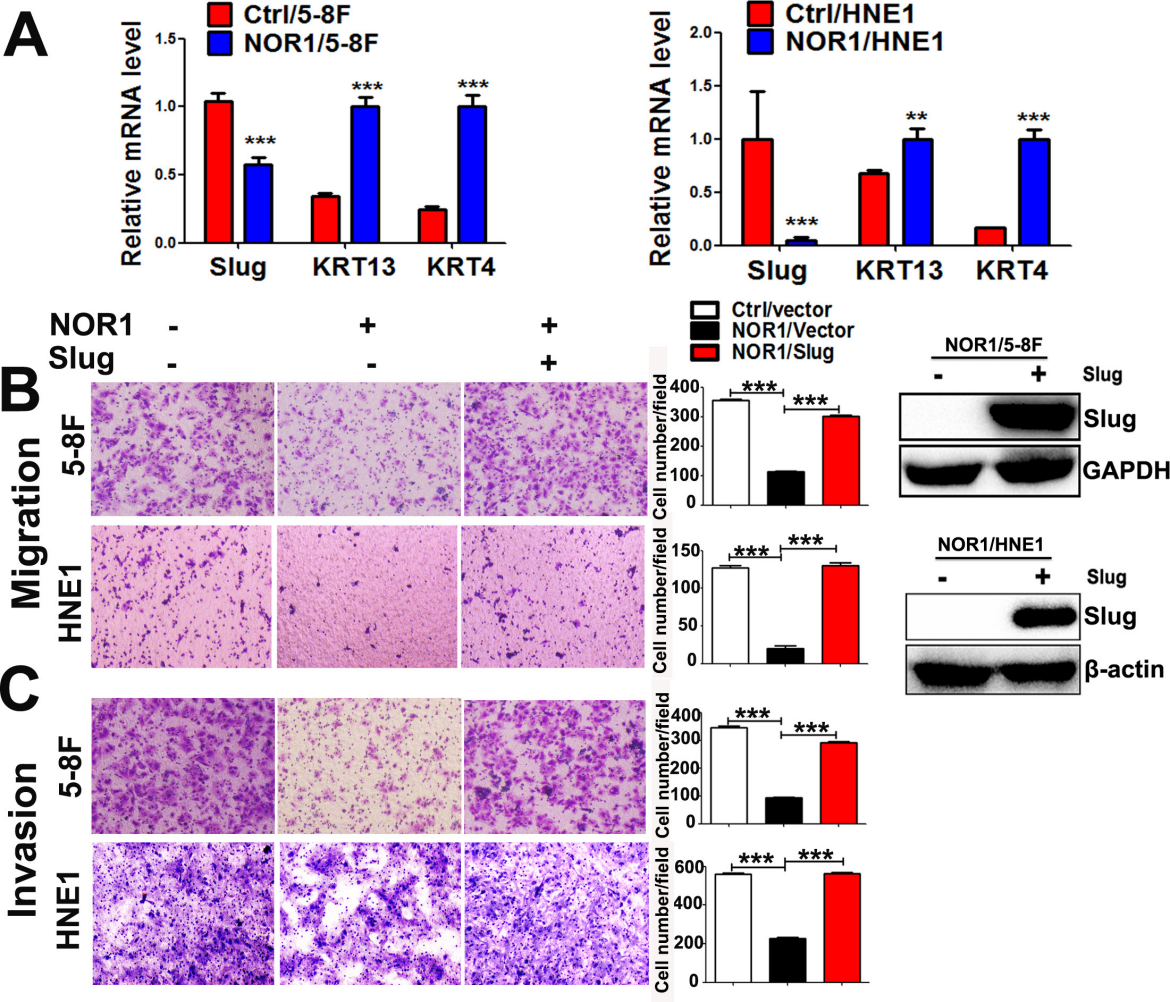
NOR1 suppresses the slug-induced EMT process and invasiveness of NPC cells (**A**) qRT-PCR analysis of the EMT transcription factor Slug and the epithelial marker keratin in NPC 5–8F, and HNE1 cells expressing control vectors pIRES or pIRES/NOR1. (**B**) Tumor cell migration assay. Ectopic expression of NOR1 suppresses migration of NPC 5-8F and HNE1 cells, but migration was rescued by concomitant transfection with a Slug expression plasmid. The graph represents summarized data of five independent experiments. Expression of exogenous Slug protein in transfected cells was determined by western blotting. (**C**) Tumor cell invasion assay. The same cells described in (B) were subjected to a tumor cell invasion assay. The graph provides summarized data of five independent experiments. **P < 0.01, ***P < 0.001 relative to the control cell data.

**Figure 4:**
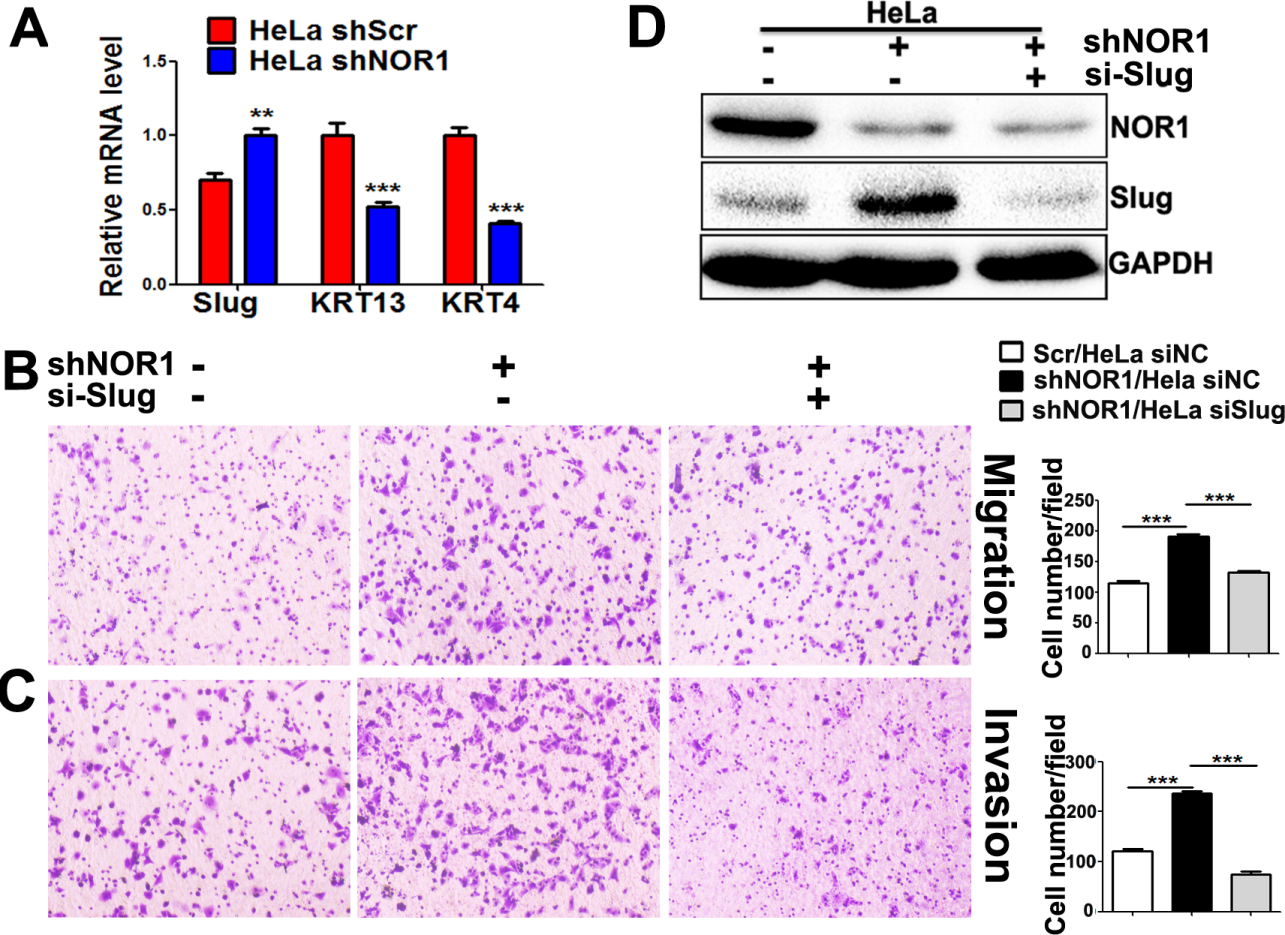
Stable silencing of endogenous NOR1 induces EMT-like properties, migration, and invasion of HeLa cells via upregulation of slug (**A**) qRT-PCR analysis of the EMT transcription factor Slug and the epithelial markers keratin in HeLa cells with or without NOR1 expression. (**B**) Tumor cell migration assay. Stable silencing of NOR1 promotes migration of HeLa cells, but migration was alleviated by concomitant inhibition of Slug. (**C**) Tumor cell invasion assay. The same cells described in B were subjected to a tumor cell invasion assay. The graph provides summarized data of five independent experiments. (**D**) Scrambled shRNA or NOR1 shRNA along with Slug siRNA-transfected HeLa cells were subjected to western blotting. **P < 0.01, ***P < 0.001 compared to the control cells.

Lost expression of NOR1 in NPC tissues and its inverse correlation with Slug expression led us to hypothesize that NOR1 might function as a novel antagonist of EMT and tumor invasion. We then examined the effects of NOR1 on the EMT-like properties of tumor cells *in vitro*. These results were consistent with our previous report [26]; qRT-PCR showed that ectopic expression of NOR1 suppresses Slug mRNA but enhances keratin 4 expression and keratin 13 mRNA levels in NPC cells (Figure 3A). Functionally, ectopic expression of NOR1 suppresses both migration and invasion of NPC 5–8F and HNE1 cells *in vitro*, while concomitant transfection with a Slug-expressing plasmid rescued NPC cell aggressiveness (Figure 3B and 3C). Conversely, stable silencing of NOR1 induced *slug* but reduced keratin 4 and keratin 13 mRNA levels in HeLa cells (Figure 4A). Consequently, it can be concluded that loss of NOR1 in HeLa cells leads to increased aggressiveness in HeLa cells (Figure 4B and 4C). However, concomitant silencing of Slug resulted in the reduction of both migration and invasion (Figure 4B and 4C and 4D). These data indicate that NOR1 acts as an antagonist of the EMT and tumor aggressiveness primarily through inhibition of Slug transcription factors.

### NOR1 induces chromatin remodeling by disturbing balance of slug-associated H3K9 acetylation and tri-methylation

**Figure 5:**
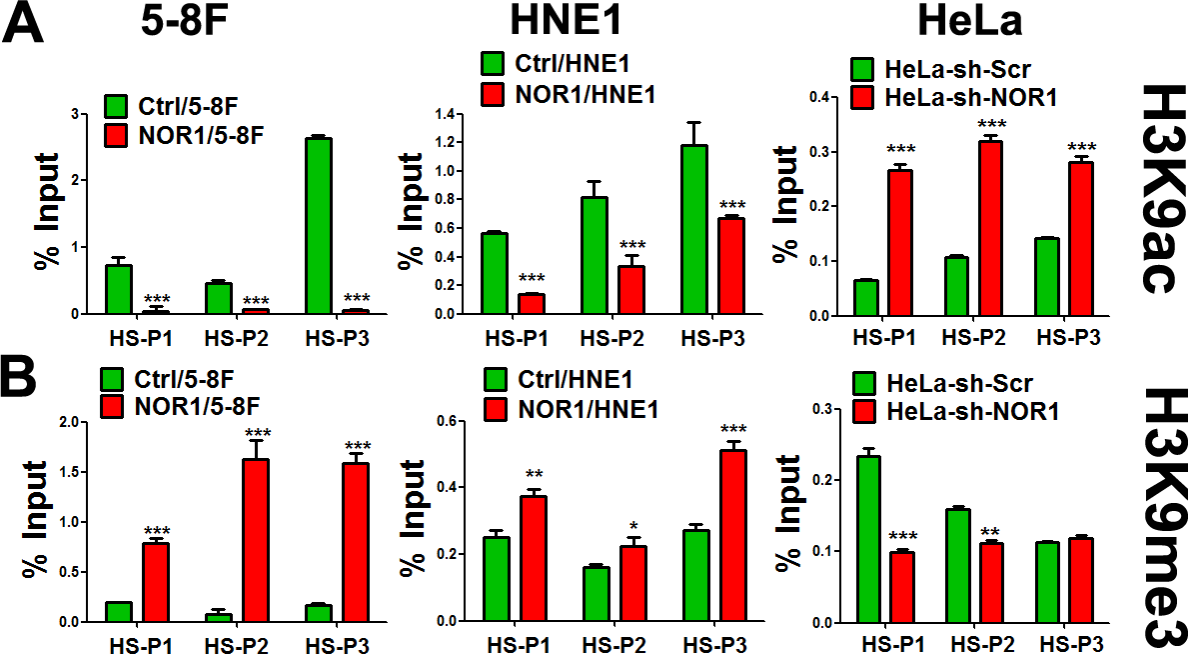
NOR1 expression induces chromatin remodeling of slug promoter regions in cancer cells ChIP-qPCR assay: tumor cells with or without NOR1 expression were subjected to ChIP with antibodies specific to acetyl-H3K9 (H3K9Ace), trimethyl-H3K9 (H3K9Me3), or control IgG, followed by amplification with three PCR primers sets(HS-P1, HS-P2 and HS-P3) specific for the slug promoter regions. Data show the representative results from three independent experiments. *P < 0.05, **P < 0.01, ***P < 0.001 compared to the control cells.

The induction of EMT is accompanied by a dynamic reprogramming of the epigenome; this reprogramming involves changes in DNA methylation and several post-translational histone modifications [7]. We analyzed molecular mechanisms underlying the inhibition of Slug by NOR1. Because histone modifications play critical roles in gene silencing through chromatin remodeling, we tested whether NOR1-mediated expressional decrease of Slug was accompanied by changes in the chromatin structure of the region. ChIP analysis of the *slug* promoter and 3-UTR was performed with H3K9 antibodies. Subsequent qPCR performed using the precipitate as the template showed decreased Slug-associated acetylation of H3K9 (H3K9Ace; active chromatin marker) and concurrent increased tri-methylation at the same residue (H3K9Me3; repressed chromatin marker) in response to NOR1 expression in both HNE1 and 5–8F cells (Figure 5A and 5B). Conversely, an increase in Slug-associated H3K9 acetylation and a decrease H3K9 tri-methylation levels occurred in NOR1 stable knockdown HeLa cells (Figure 5A and 5B). Thus, our data suggest that repression of Slug expression by NOR1 is accomplished by the alteration of Slug-associated chromatin remodeling.

**Figure 6:**
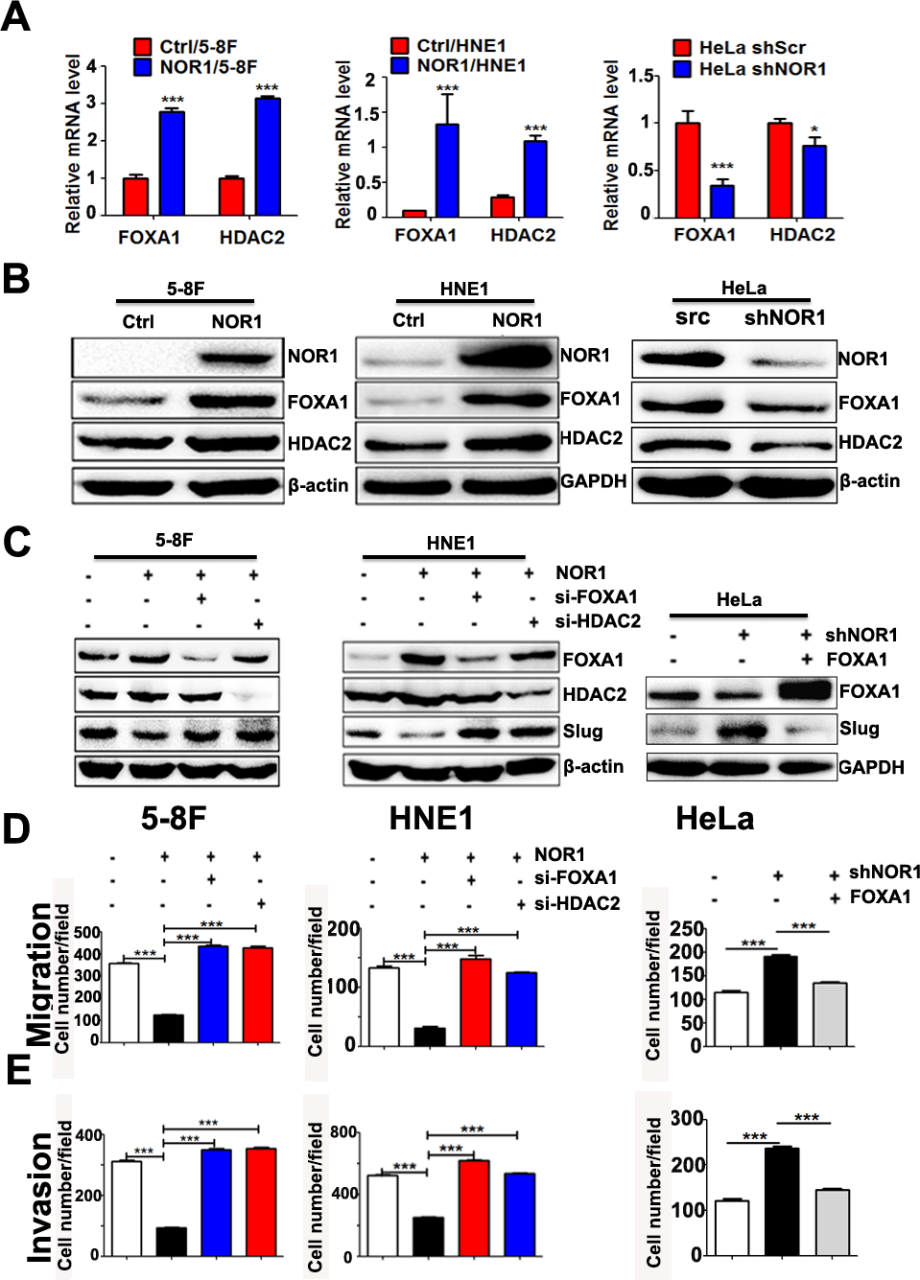
NOR1 inhibition of slug expression and malignant behaviors of cancer cells are dependent on FOXA1 and HDAC2 (**A**) qRT-PCR assay; ectopic expression of NOR1 or silencing of endogenous NOR1 leads to upregulation or downregulation of FOXA1 and HDAC2 mRNAs in cancer cells, respectively. (**B**) Western blot assay; ectopic expression of NOR1 or silencing endogenous NOR1 leads to up-regulation or down-regulation of FOXA1, HDAC2 proteins in cancer cells respectively. (**C**) Western blot assay; silencing either FOXA1 or HDAC2 using siRNAs results in recovery of Slug protein in NOR1-expressing NPC cells. Conversely, ectopic expression of FOXA1 protein leads to decreased Slug protein levels in NOR1-silenced HeLa cells. (**D**) Tumor cell migration assay; silencing either FOXA1 or HDAC2 using siRNAs rescues the migration of NOR1-expressing NPC 5–8F and HNE1 cells, while ectopic expression of FOXA1 suppresses the migration of NOR1-deficient knockdown HeLa cells. (**E**) Tumor cell invasion assay. The same cells described in (D) were subjected to a tumor cell invasion assay. The graph provides summarized data of five independent experiments. *P < 0.05, ***P < 0.001 compared to the control cells.

### Suppression of slug expression and malignant behaviors of cancer cells by NOR1 is dependent on modulation of FOXA1 and HDAC2

Because NOR1 was found to be a primarily cytoplasmic protein, NOR1 likely indirectly regulates Slug-associated histone modification and transcription by interacting with other transcription factors (TFs) and histone modification enzymes such as HDACs. As mentioned above, NOR1 mRNA levels are directly correlated with FOXA1 mRNA levels, which led us to speculate that FOXA1 might be one of the potential TFs involved in NOR1-mediated reverse of the EMT. We then measured the expression level of FOXA1 and HDAC2 in NPC cells and HeLa cells with and without NOR1 expression. A qRT-PCR assay showed both FOXA1 and HDAC2 mRNA levels were increased after stable NOR1 expression in HNE1 and 5–8F cells (Figure 6A and 6B). Conversely, silencing endogenous NOR1 protein expression led to a decrease in both FOXA1 and HDAC2 mRNA levels and protein levels in HeLa cells (Figure 6A and 6B). However, we did not observe significant changes in the mRNA expression of the histone methyltransferase genes *G9a*, *Suv39h1*, and *Suv39h2* between NOR1-deficient or NOR1-expressing cells (Supplementary Figure 1).

Ectopic expression of NOR1 resulted in a decrease of Slug protein levels, while silencing either FOXA1 or HDAC2 in NOR1-expressing NPC 5–8F and HNE1 cells rescued the Slug protein levels (Figure 6C). Stable silencing of endogenous NOR1 led to an increase of Slug protein levels in HeLa cells. However, on transfection with a FOXA1 expression plasmid and a NOR1 shRNA-expressing vector, Slug protein levels decreased in HeLa cells (Figure 6C). Functionally, silencing either FOXA1 or HDAC2 in NOR1-expressing NPC 5–8F and HNE1 cells rescued NPC cell aggressiveness (Figure 6D and 6E). Concomitant transfection with the FOXA1 expression plasmid and a NOR1 shRNA-expressing vector reduced HeLa cells aggressiveness (Figure 6D and 6E). These data indicate that NOR1-mediated Slug inhibition and reversal of the EMT process are dependent on the upregulation of either FOXA1 or HDAC2.

### Both FOXA1 and HDAC2 directly suppress *slug* transcription in NPC cells

**Figure 7:**
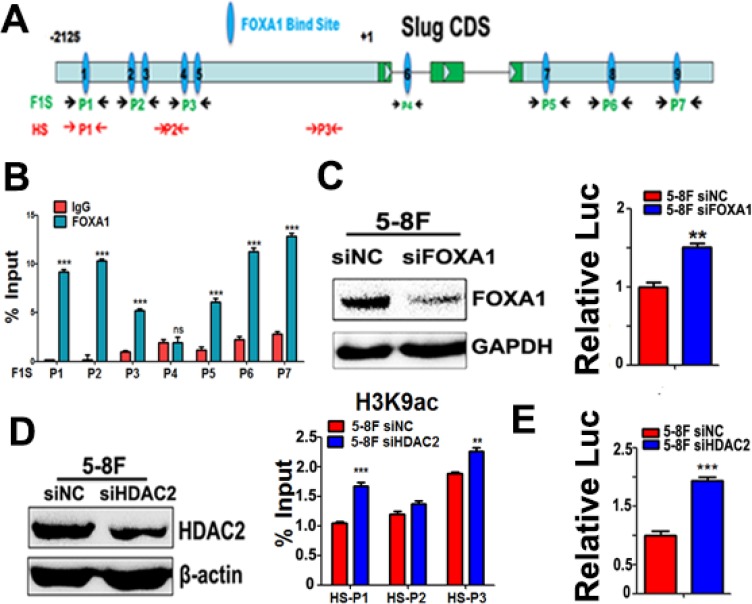
Either FOXA1 or HDAC2 negatively regulates slug transcription (**A**) A schematic illustration of slug gene structure and the potential binding sites for different primer sets. Seven primer sets named as F1S-P1~ F1S-P7 were used for validation of FOXA1 binding sites on Slug promoter regions and downstream 3-UTR region. Three primer sets named as HS-P1~ HS-P3 were used for amplification slug gene DNA fragments associated with acetylated-H3K9. (**B**) ChIP was performed by using anti-FOXA1 antibody, and qPCR following ChIP was performed to identify the binding sites in the Slug promoter and downstream 3-UTR region in 5–8F cells by using seven primer sets(F1S-P1~ F1S-P7). Normal rabbit IgG was used as a negative control. The quantification of the band intensity of PCR products is shown on the right side. (**C**) Activation of slug promoter activity by co-transfection with FOXA1-targeted siRNAs in 5–8F cells. FOXA1 protein levels were measured by western blotting. Relative luciferase activities of the slug promoter are shown. Values are the mean of three independent assays, and the error bar is the standard deviation of the mean. (**D**) ChIP was performed using anti-acetylated-H3K9 antibody, and qPCR following ChIP was performed to compare the changes of slug promoter-associated H3K9-acetylation levels after silencing HDAC2 in 5-8F cells. Increased slug promoter-associated H3K9-acetylation levels was observed by silencing of HDAC2 in 5–8F cells. HDAC2 protein levels were measured by western blotting. (**E**) Activation of slug promoter activity by co-transfection with a HDAC2-targeted siRNAs in 5–8F cells. Relative luciferase activities of the slug promoter are shown. Values are the mean of three independent assays, and the error bar is the standard deviation of the mean. **P < 0.01, ***P < 0.001 compared to the control cells.

To analyze whether FOXA1 directly regulates transcription of *slug*, potential FOXA1 binding sites were mapped in the *slug* promoter/enhancer shown in Figure 7A. In 5–8F cells, ChIP analysis identified three FOXA1-bound regions (F1 and F3) within the promoter region of *slug* and three FOXA1-bound regions within the 3′-UTR regions (F5 and F7) of the *slug* locus (Figure 7B). FOXA1 could not bind to the predicted binding site in the first intron (F4) of the *slug* gene. To determine the effect of FOXA1 binding on *slug* promoter activities, we performed a *slug* promoter-induced luciferase activity assay, and our data showed that *slug* promoter activities increased in FOXA1 knockdown 5–8F cells (Figure 7C). This suggests that Slug is a novel direct repression target of FOXA1. We also found that Slug-associated H3K9-ace was controlled by histone acetyltransferase HDAC2. Silencing of HDAC2 in 5–8F cells induced an increase of Slug-associated H3K9-ace (Figure 7D), followed by activation of the *slug* promoter (Figure 7E). Thus, our data confirmed that both FOXA1 and HDAC2 directly suppress Slug transcription in NPC cells. We further showed that transiently silencing FOXA1 or HDAC2 in NPC cells was efficient in initiating the loss of epithelial traits, while enhancing NPC aggressiveness (Supplementary Figure 2). Collectively, these findings indicate that FOXA1 and HDAC2 are essential in maintaining the epithelial phenotype in NPC.

## DISCUSSION

Nasopharyngeal carcinoma is highly metastatic in comparison to other head and neck tumors. The epithelial-to-mesenchymal transition is considered a major metastasis-promoting mechanism in human carcinomas. Early observations have identified several EMT-promoting pathways such as those involving BMI-1 oncogene activation [28] and EBV oncoprotein LMP1 [29], while relatively little attention has been paid to EMT-suppressing pathways in NPC. In this study, we report a previously unrecognized role for the NOR1-FOXA1/HDAC2-Slug network in the regulation of EMT and invasiveness in NPC cells.

Several transcription factors have proven to be powerful regulators of EMT regulators, including members of the Snail (SNAIL1, Slug) and basic helix-loop-helix (bHLH) families, and two double zinc finger and homeodomain (ZEB) factors [30]. Although such EMT regulators are thought to function in a redundant manner, several recent studies suggest unique functions for Slug [31, 32]. In this study, from an NPC GEO dataset, we found that Slug mRNA levels continuously increased in NPC samples, independently of the clinical stages of NPC. However, Snail1, Twist1 and Twist 2 showed no differences between NPC tissues and their normal counterparts. These differences may suggest that Slug might be more relevant than Snail or Twist in the generation of NPC cells with an EMT phenotype. Our hypothesis is also supported by others' observation that Slug has been reported to play more important roles than Snail in generating a breast cancer stem cell phenotype [33]. Pilot works showed that Twist1 protein was overexpressed in NPC samples from progressive stage patients [14]; consequently, overexpression of TWIST1 seems to be a late event during NPC progression. It has been proposed that the EMT could occur during the very early stages of tumorigenesis and is a prerequisite property for invasiveness and metastasis. As NPC is well characterized in its early tendency to locally spread to the parapharyngeal space in an early stage, another explanation should be drawn, namely, that Slug and Twist proteins seem to act at different stages and support distinct tumor invasion modes during NPC progression.

We observed that Slug mRNA levels are inversely correlated with the mRNA levels of epithelial marker keratin 4. This makes sense, as keratin 4 was shown to be a repressive target of Slug. Another well-documented epithelial marker, E-cadherin, showed no significant differences between normal nasopharynx epithelia and NPC biopsy samples. This could be explained in that Slug expression is not always associated with downregulation of E-cadherin in many cancers [34]. Thus, the expression patterns of Slug in NPC progression suggested unique characteristics of EMT-like properties during NPC progression.

A previous study indicated that NOR1 acts as an antagonist in metastasis during NPC progression [26]. In this study, unbiased analysis of a public GEO profile derived from microdissected NPC tissues confirmed that NOR1 mRNA expression markedly decreased in NPC samples. More importantly, NOR1 mRNA levels are directly correlated with keratin 4 mRNA levels, but inversely correlated with Slug mRNA levels (marginally significant), which strongly implies that NOR1 might be an antagonist of the Slug-induced EMT process. Furthermore, NOR1 suppresses Slug expression and the EMT-like properties of NPC cells and HeLa cells, which is consistent with our previous report [26]. Our *in vitro* and *ex vivo* data collectively indicate that NOR1 functions as an antagonist of the Slug-induced EMT process during NPC progression.

FOXA1, a member of the forkhead transcription factor family, is critical for both early embryonic development and late or end stage epithelial differentiation. Several studies suggested that FOXA1 functions as an important antagonist of the EMT in different cancer types [18, 19], but its role in NPC is not well characterized. In this study, unbiased public GEO dataset data derived from microdissected NPC tissues showed a sharp decrease of FOXA1 mRNA in NPC samples, which was also confirmed by immunohistochemical staining. In this study, we confirmed that FOXA1 binds to the promoter and 3-UTR region of *slug* and impairs activation of the promoter. This observation is in agreement with previous reports that FOXA1 directly represses transcription of *slug* by binding the *slug* promoter in those FOXA1 binding sites [16, 35]. Transient silencing of FOXA1 in NPC cells initiated loss of epithelial traits and accelerated NPC cell migration and invasion (Supplementary Figure 2). In NPC samples, FOXA1 mRNA levels are directly correlated with those of NOR1. Our *in vitro* data also showed that FOXA1 mRNA and protein levels increased in NPC cells with NOR1 ectopic expression, while they sharply decreased in NOR1-silenced HeLa cells. NOR1-mediated inhibition of Slug seems to be dependent on FOXA1, as knocking down FOXA1 relieves the inhibition of NOR1 on Slug. Thus, our *in vitro* and *ex vivo* data collectively indicate that NOR1 cooperatively suppresses Slug-induced EMT with FOXA1. Currently, the mechanism underlying NOR1-mediated upregulation of FOXA1 in NPC cell lines is not known and this needs to be studied further.

The expression of FOXA1 mRNA showed no correlation with *slug* mRNA levels in NPC samples. Our results are quite heterogeneous regarding this matter and are not in accordance with our *in vitro* data or others' findings [16, 19], where the association is clear. It is not clear, from this set of data, whether FOXA1 is in fact responsible for Slug upregulation in NPC biopsy samples. This suggests that FOXA1 is not the only transcription factor responsible for Slug regulation *in vivo*. However, one must consider that gene regulation is much more complex *in vivo* and that *in vitro* models are limited and do not always represent the *in vivo* situation [34].

Covalent modification of histones plays important roles in regulating transcription, genome integrity, and epigenetic inheritance. Gene activation involves a competition between TFs and histones for DNA binding [12]. Post-translational modifications of histones regulate the access of TFs to DNA. In this study, H3K9 de-acetylation and concomitant tri-methylation at the *slug* promoter region occurred in NOR1-expressing NPC cells. Our data indicate that HDAC2 is at least partially responsible for de-acetylation at *slug* promoter-associated H3K9 in NPC 5–8F cells, which in turn leads to a decrease of *slug* promoter activities. These data are consistent with another report that HDAC2 binding of the *slug* promoter might be responsible for the suppression of Slug production [36]. Silencing HDAC2 alone in NPC cells initiated loss of epithelial traits and accelerated the migration and invasion of NPC cells (Supplementary Figure 2). Thus, our data, in light of other data, clearly indicate that *slug* transcription is controlled by HDAC2. Silencing HDAC2 also relieved *slug* repression by NOR1 in NPC cells. NOR1 expression-induced alteration of Slug-associated histone modification is thought to be at least partially mediated by HDAC2, as evidenced by relief from *slug* repression of NOR1 via HDAC2 silencing. As the TSG NOR1 is silenced by DNA hypermethylation in NPC and hematological malignancies [13, 22, 24], our results also suggest cooperation between DNA methylation and histone modification in the EMT process.

It is well documented that acetylation and methylation of H3K9 are mutually exclusive [37]. In contrast to H3K9 acetylation, which is generally associated with active transcription, methylation at H3K9 usually leads to transcriptional repression. It has been reported that *G9a* is responsible for H3 Lys9 mono- and dimethylation, whereas the gene products of *Suv39h1* and *Suv39h2* are responsible for the majority of H3 Lys9 trimethylation [38]. Although expression of *G9a*, *Suv39h1* and *Suv39h2* was unaffected by NOR1 expression (Supplementary Figure 1), NOR1 expression led to alteration of tri-methylated H3K9 levels in NPC cells and HeLa cells. However, this does not rule out the possibility that NOR1 expression might affect the level of enzyme activity of those three HMTases. Another explanation is that one mechanism that HDAC inhibition in NOR1-knockdown cells might enhance *slug* transcription is through secondary suppression of histone H3K9 trimethylation. This might be supported by previous observations that inhibition of histone deacetylases (HDACs) with sodium butyrate (NaB) resulted in decreased H3K9 dimethylation in the hippocampus following contextual fear conditioning [39].

**Figure 8:**
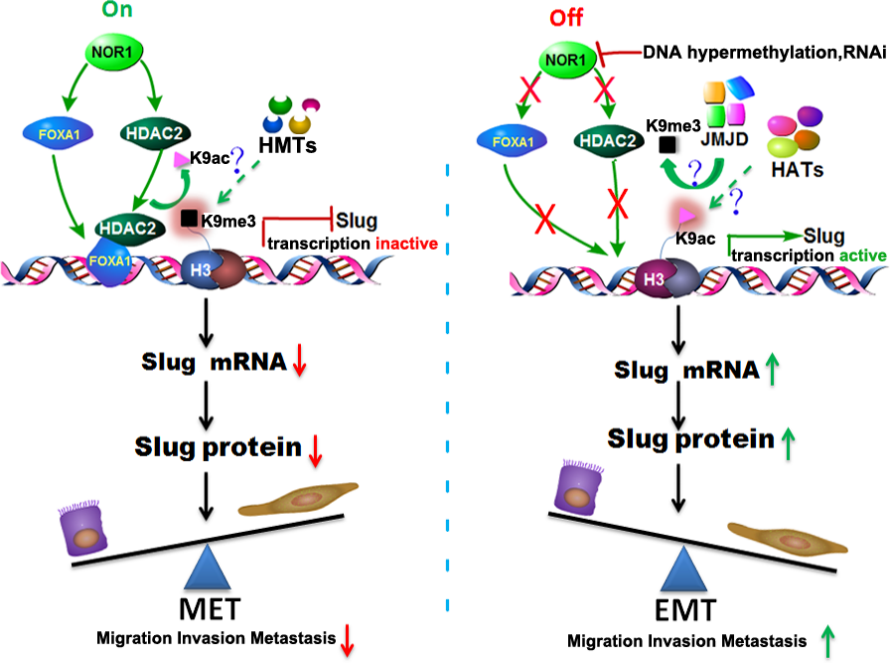
Schematic illustration of the NOR1-FOXA1/HDAC2-SLUG regulatory network in the EMT process of NPC In normal nasopharyngeal epitheliums, high levels of NOR1 expression lead to optimal expression of FOXA1; FOXA1 then translocates to the nucleus, recruits HDAC2, and binds to the slug promoter region, resulting in de-acetylation and secondary tri-methylation of Slug-associated H3K9. The transcription of Slug is repressed and Slug protein levels are decreased, so the cells are maintained as epithelial cells. During NPC development, NOR1 is silenced by DNA hypermethylation or deletions, which in turn results in a decrease in the expression of FOXA1 and HDAC2, thus removing the inhibitory effect on the transcription of slug. The transcription of slug is activated and Slug protein production is upregulated, subsequently leading to the Slug-induced EMT process. HMTs, histone methyl transferases. JMJD, Jumonji domain–containing protein histone demethylases. HATs, histone acetyltransferases.

Based on our findings from *ex vivo* human NPC specimens and *in vitro* studies in NPC cell lines, we speculate that NOR1 cooperates with FOXA1 and HDAC2 to play a critical role in the suppression of the Slug-induced EMT process during NPC progression. We propose a model where the NOR1 and FOXA1 factors are constitutively expressed in normal nasopharynx epithelia and in dysplastic epithelial tissue where they maintain optimal keratin levels, thus suppressing EMT by facilitating binding of the FOXA1-HDAC2 complex to the *slug* promoter. When NOR1 function is “off” (e.g., silenced by DNA hypermethylation during NPC progresses [24]), NOR1 protein levels decrease, which secondarily leads to dysfunction of the FOXA1-HDAC2 complex, thus relieving the inhibitory effect on the Slug-induced EMT process (Figure 8). In summary, our recent data have shown that NOR1 antagonizes the EMT process in NPC cells, mainly through suppressing Slug and inducing epithelial keratin expression. NOR1 suppression of the Slug transcript is associated with H3K9 de-acetylation and concomitant H3K9 tri-methylation. The repressive effect of NOR1 on Slug is dependent on upregulation of transcription factors FOXA1 and HDAC2. Together these data lead us to conclude that dysfunction of the NOR1-FOXA1/HDAC2-Slug network is an essential step in the EMT program during NPC progression.

## MATERIALS AND METHODS

### Dataset

The global gene expression profile data set GSE12452 (http://www.ncbi.nlm.nih.gov/geo/query/acc.cgi?acc=GSE12452) was collected from Gene Expression Omnibus (GEO). The dataset contains 31 NPC and 10 normal nasopharyngeal tissue samples examined with a Human Genome U133 Plus 2.0 Array (HG-U133 Plus 2) from Affymetrix [27]. All the samples were histologically confirmed and processed by laser-captured microdissection to enrich the epithelial cells before total RNA extraction.

### Tumor tissue samples and immunohistochemistry

A cohort of 33 subjects with NPC, as well as non-cancerous nasopharyngeal epithelial (NPE) control subjects, were recruited between January 2001 and October 2004 from the Xiangya Hospital, and their tissue samples were used for immunohistochemical analysis of NOR1, FOXA1, and Slug expression. Immunohistochemistry was performed according to methods described in previous studies [24, 40]. This study was approved by the Institute Research Ethics Committee for use of clinical samples and each patient signed a consent form to participate in the study. A polyclonal anti-NOR1 antibody, polyclonal anti-slug antibody was obtained from Proteintech Group (Chicago, IL, USA). A polyclonal anti-FOXA1 antibody was obtained from Epitomics Inc(Burlingame, CA, USA).

### Cell lines and culture

The NOR1-stable transfected undifferentiated NPC-derived cell lines HNE1 and 5–8F were maintained as previously reported [25, 26]. Cells were grown in RPMI 1640 medium supplemented with 10% fetal bovine serum (FBS) and penicillin/streptomycin (GIBCO, Grand Island, NY) in a humidified incubator at 37°C with 5% CO_2_ and 95% air. The cervical cancer cell line HeLa (ATCC CRL-2) was obtained from ATCC (Manassas, Virginia, USA) and cultured in Dulbecco's modified Eagle's medium (DMEM) containing 10% FBS, 100 U/ml penicillin, and 100 mg/ml streptomycin in a humidified incubator at 37°C with 5% CO_2_ and 95% air.

### Construction of *slug* promoter and iuciferase activities assay

The upstream regulatory regions (from -34- to -2125 bp) of the *slug* gene were amplified from healthy human peripheral blood DNA by PCR with a high-fidelity thermal polymerase (Platinum *Pfx* DNA polymerase, Life Technologies, Gaithersburg, MD, USA). PCR fragments were digested with *Mlu*I/*Xho*I and linked to the luciferase-based promoter-less plasmid – pGL3-Enhancer Vector (Promega, Madison, WI, USA) to construct plasmids. For the luciferase-based assay, the *slug* promoter activities were tested by transient transfection of 1 μg of plasmid DNA into NPC cells using Lipofectamine 2000 Transfection Reagent (Life Technologies, Gaithersburg, MD, USA). Results were normalized against *Renilla* luciferase activity. At least three independent assays were performed.

### siRNA, shRNA and gene transfection

The siRNAs for knockdown of human FOXA1 and HDAC2 and the nonspecific siRNAs (scrambled sequences) were purchased from GenePharma (Shanghai, China) and used for transfection into NPC cells using Lipofectamine^®^ RNAiMAX Reagent (Invitrogen, Carlsbad, CA) according to the manufacturer's protocol. The Slug expression vectors were purchased from GeneCopoeia and transfected into NPC cells using Lipofectamine 2000 Transfection Reagent. For stable knock down of endogenous NOR1, a shRNA lentivirus targeting NOR1 or nonspecific lentivirus was prepared and used to infect HeLa cells. Infected cells with GFP expression were sorted by fluorescence-activated cell sorting (FACS). The siRNAs or shRNA sequences used in this study are listed in Table S1.

### RNA isolation and real-time reverse transcription PCR (qRT-PCR)

Total RNA extraction was performed as described previously [28] by using TRIzol Reagent (Invitrogen, San Diego, CA). For reverse transcription, 1 mg of total RNA sample treated with DNaseI (Roche Diagnostics, Rotkreuz, Switzerland) was reverse-transcribed into cDNA using the M-MLV reverse transcriptase according to manufacturer instructions (Promega, Madison, WI). Real-time PCR using SYBR Green I technology was then performed using a CFX96 Touch™ Real-Time PCR Detection System (BioRad). The PCR primers used in this study are listed in Table S2.

### Chromatin immunoprecipitation (ChIP) and qPCR

For ChIP analysis, cells grown on a 10-cm plate were processed as described in the ChIP Assay kit protocol from Millipore (17–295). The chromatin was immunoprecipitated with the following antibodies: anti-FOXA1 (Epitomics, 3333–1), anti–acetyl-histone H3K9 (Millipore, 17–658), and anti–trimethylhistone H3K9 (Millipore, 17–10242). The precipitated DNA fragments were measured by qPCR under the conditions described above. Primers specific to each segment of interest are listed in Table S2.

### Protein extraction and western blot

Total cellular protein was prepared from the cultured cells using a lysis buffer (Beyotime, Jiangsu, China). Equal protein loads from different extracts were separated by SDS-PAGE and transferred onto a PVDF membrane (Millipore, Billerica, MA). The membranes were immunoblotted with the following antibodies: polyclonal anti-Slug, (Cell Signaling Technology, Danvers, MA); polyclonal anti-FOXA1, anti-α-tubulin antibody (Epitomics, Burlingame, CA); polyclonal anti-keratin 4, anti-keratin 13 antibodies, anti-NOR1 antibody, anti-GAPDH antibody (Proteintech Group, Chicago, IL); anti-β-actin antibody (Santa Cruz Biotechnology, Santa Cruz, CA); and polyclonal anti-HDAC2 (ABZOOM BIOLABS, Dallas, TX, USA). The methods used were in accordance with those reported in previous studies [24, 40].

### Transwell migration and invasion assays

Tumor cells migration or invasion assays were performed according to our previous studies [26]. Briefly, cell suspensions in serum-free medium were seeded onto 8 μm-pore Transwell inserts (Corning-Costar, Cambridge, MA) at a density of 50,000–100,000 cells/well, then the inserts were held in a lower chamber with 600 μl culture media containing 15% FBS. Transwells were incubated for 6–24 h at 37°C. Cells on the inside of the Transwell inserts were removed with a cotton swab; then, cells that migrated to the lower surface of the membrane were fixed and stained. Photographs of five random fields were taken, and the cells were counted to calculate the average number of cells that had transmigrated. For the tumor cell invasion assay, the membrane was pre-coated with 15 ml Matrigel (BD Biosciences, Bedford, MA), and the rest of the method was identical to the tumor cell migration assay except for cell incubation, which was conducted for 24 to 48 h at 37°C.

### Statistical analysis

Differences in quantitative variables between groups were analyzed by the Student's *t* test. Combined strategies were used to analyze the datasets (GDS3341/213139_at/Slug, GDS3341/219480_at/SNAI1, GDS3341/213943_at/Twist1, GDS3341/229404_at/Twist2, GDS3341/20 1426_s_at/VIM, GDS3341/201131_s_at/E-cadherin, GD S3341/213240_s_at/KRT4, GDS3341/207935_s_at/KR T13, GDS3341/227359_at/NOR1 and GDS3341/204667_at/FOXA1) gained from the NPC GEO profiles GSE12452. The expression matrix were downloaded and processed by statistical methods. Briefly, Log (base 2) expression measures for each probe set were computed using robust multiarray average according to a previous report [41]. The values of *Slug, Snail, Twist1, Twist2, VIM, E-cadherin, KRT4, KRT13, NOR1* and *FOXA1* genes expression in the 31 NPC and 10 normal nasopharyngeal tissue samples were calculated by single tail test. The Pearson χ^2^ was used to analyze the association of NOR1 expression with clinicopathological characteristics using the SPSS 13.0 software package (SPSS, Chicago, IL). A value of *P* < 0.05 was considered statistically significant.

## SUPPLEMENTARY MATERIALS FIGURES AND TABLES


