# Supplementary material for: Significance of the NOR1-FOXA1/HDAC2-Slug regulatory network in epithelial-mesenchymal transition of tumor cells
Source: Oncotarget. 2016 Feb 27;7(13):16745–59. doi: 10.18632/oncotarget.7778 (PMC4941348; doi:10.18632/oncotarget.7778)
Supplement: Supplementary file 1 [file oncotarget-07-16745-s001.pdf]

Significance of the NOR1-FOXA1/HDAC2-Slug regulatory network in epithelial-mesenchymal transition of tumor cells

Supplementary Materials

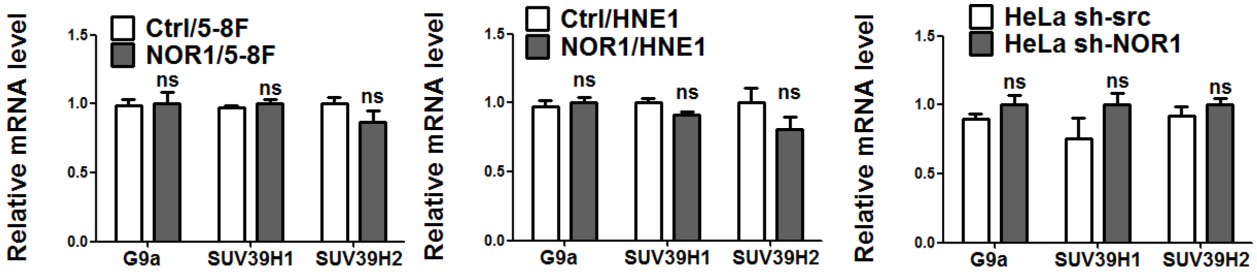

Supplementary Figure S1: mRNA levels of histone methyltransferase genes in NOR1-deficient or NOR1-expressing tumor cells were measured by qRT-PCR. The data showed that ectopic expression of NOR1 in NPC 5-8F and HNE1 cells does not affect the mRNAs level of *G9a*, *Suv39h1* or *Suv39h2*. As expected, stable silencing of NOR1 in HeLa cells also did not affect the mRNA levels of *G9a*, *Suv39h1* and *Suv39h2*.

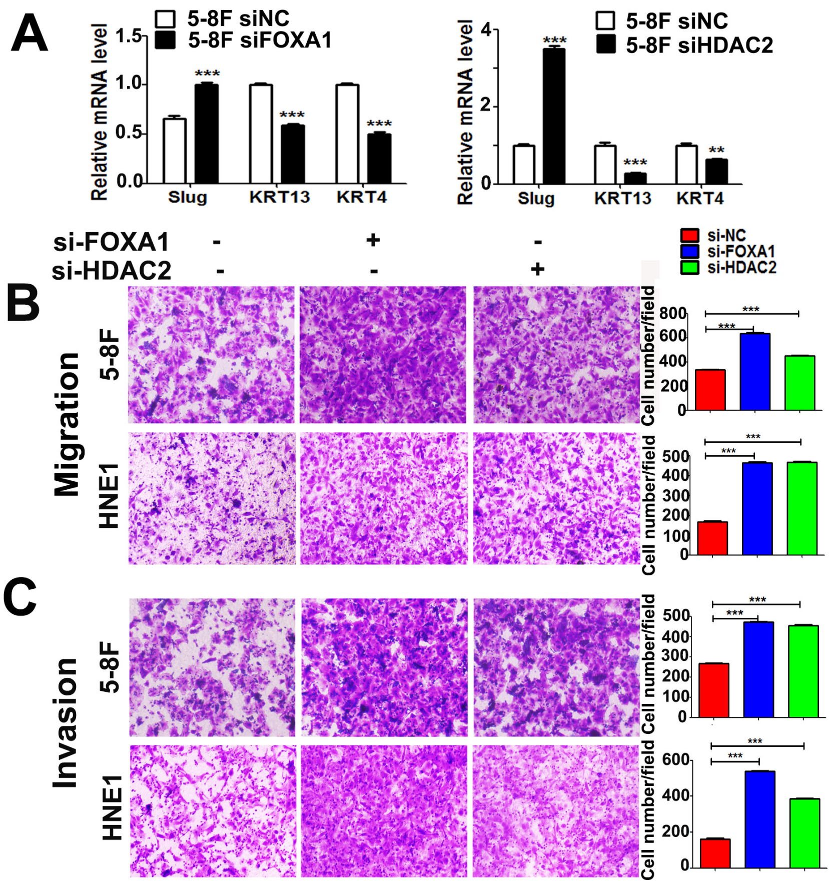

Supplementary Figure S2: Transiently silencing FOXA1 or HDAC2 initiates the EMT process in NPC cells. (**A**) NPC 5-8F cells were transiently transfected with siRNA against either FOXA1 or HDAC2. Expression of FOXA1, HDAC2, keratin 4, keratin13 and Slug was examined by qRT-PCR 48 hours after siRNA transfection. (**B**) Silencing of FOXA1 or HDAC2 results in increased cell migration in Transwell migration assays. Cells that migrated from serum-free to serum-containing medium within 6 hours were stained with crystal violet (micrographs) and counted (bar graphs). Quantitation is from five random fields from each membrane, and averaged from triplicates of three independent experiments. Error bars represent SEM. (**C**) Transwell invasion assays. The same cell types were subjected to a tumor cell invasion assay. The graph provides summarized data of five independent experiments. $^{**}P < 0.01$, $^{***}P < 0.001$ as compared to the control cells.

Supplementary Table S1: siRNA or shRNA sequences used in this study

	sense 5′–3′
Scrambled siRNAs	UUCUCCGAACGUGUCACGUTT
FOXA1 siRNA	GAGAGAAAAAAUCAACAGC
HDAC2 siRNA	GCCUCAUAGAAUCCGCAUG
Slug siRNA	AUCAGAAUGGGUCUGCAGAUGAGCC
Scrambled shRNAs	TTCTCCGAACGTGTCACGT
NOR1 shRNA	GAACAUCCCGGGAGACAAG

Supplementary Table S2: Primers used in this study

Genes	5′—3′	
**Primers used in ChIP**		**Location**
F1S-P1 F:	CACCCTCGGATACCTGCTGA	−1976–−1755
F1S-P1 R:	TCCTTGTTTCACTCTACACAGTC	
F1S-P2 F:	ACTGTGTAGAGTGAAACAAGGA	−1776 –−1642
F1S-P2 R:	GGGTTTATGAGAGCAGGAGT	
F1S-P3 F:	TTAGGAAATCTGTGAGTGCC	−1410–−1240
F1S-P3 R:	TCTAACAGGTGCTGGAGG	
F1S-P4 F:	GTTGAGGCTCTCCTTCCT	693 – 871
F1S-P4 R:	CCATTTAGGAGGGCATAC	
F1S-P5 F:	AAATGCATACCACAAATGCAA	3436 – 3569
F1S-P5 R:	ACAACATCTCAGTTTCATACAG	
F1S-P6 F:	TCACACTTTCTGGGTGCA	3913 – 4054
F1S-P6 R:	ACTGTTACAGTGTGTAGGTA	
F1S-P7 F:	ATGATAGTAAGGAGAGGTCC	5512 – 5628
F1S-P7 R:	CTATACATGCACATTGTACC	
HS-P1 F:	CACCCTCGGATACCTGCTGA	−1976–−1755
HS-P1 R:	TCCTTGTTTCACTCTACACAGTC	
HS-P2 F:	GTGTGTGGAGAAATCGAA	−1559–−1391
HS-P2 R:	GGCACTCACAGATTTCCTAA	
HS-P3 F:	CAAATGACAGTTACCTCTTGC	−775–−669
HS-P3 R:	AAAGAAATGCTTTGTAGCCC	

Primers used in qPCR

FOXA1 F:	TCCAGGATGTTAGGAACTGTGA
FOXA1 R:	CCGCTCGTAGTCATGGTGTT
HDAC2 F:	CCCCTCCTCTCCTCCC
HDAC2 R:	GGGATGACCCTGTCCATAAT
Slug F:	AGATCTGCCAGACGCGAACT
Slug R:	GCATGCGCCAGGAATGTTCA
KRT4 F:	AGCCGAGAATGACTTTGTG
KRT4 R:	TGTCGCTGACATGGGTCT
KRT13 F:	TCCAGGGACTCATCAGCA
KRT13 R:	GAGGAAGGGAAACCAATCA
SUV39H1 F:	TGCCCAAATCGTGTGGTACA
SUV39H1 R:	CTGACGGTCGTAGATCTGGC
SUV39H2 F:	TCGATACTCGTCTTCCCCGA
SUV39H2 R:	CTCTGCAAGTCACAGCTCCA
G9a F:	GATTTCCACGCATCGCCTTC
G9a R:	TCTCAGAGCCACATTGGCAG
